# Biomarkers in urinary tract infections – which ones are suitable for diagnostics and follow-up?

**DOI:** 10.3205/id000068

**Published:** 2020-11-26

**Authors:** József Horváth, Björn Wullt, Kurt G. Naber, Béla Köves

**Affiliations:** 1 BKMK SZTE ÁOK Okt. Kh. Urológiai Osztálya, Kecskemét, Hungary; 2 Division of Microbiology, Immunology and Glycobiology, Lund University, Lund, Sweden; 3 Department of Urology, Technical University of Munich, Munich, Germany; 4 Jahn Ferenc Dél-pesti Kórház és Rendelőintézet, Budapest, Hungary

**Keywords:** biomarker, urinary tract infection

## Abstract

**Introduction:** Urinary tract infections (UTIs) are one of the most common infections worldwide. Under special circumstances, clinicians must rely on laboratory findings, which might have a weak predicting value, misguiding the practitioners and leading to incorrect diagnosis and overuse of antibiotics. Therefore, there is an urgent need for reliable biomarkers in UTIs.

**Methods:** We performed a literature search for biomarkers used in UTIs from January 1999 until May 2020. We used “urinary tract infection” and “biomarker” as the main key words in the PubMed, Medline and Cochrane databases. After peer review, we excluded the duplicates and identified the suitable articles, from which we collected the data and divided the available biomarkers into 5 groups: i) conventional markers; ii) promising, thoroughly studied biomarkers; iii) promising biomarkers that need further studies; iv) biomarkers of unknown significance; v) controversial, not useful markers.

**Results:** We found 131 articles, mostly from the paediatric population. Neutrophil gelatinase-associated lipocalin (NGAL) and interleukins (IL) have a leading role in diagnosing and differentiating UTIs based on a lot of observational, comparative trials. Heparin Binding Protein (HBP), Lactoferrin (LF), Heat-Shock Protein-70 (HSP-70), Human Defensin-5 (HD-5), Lipopolysaccharide Binding Protein (LBP) and mass spectrometry studies are promising, but confirming data are lacking. The measurable components of the innate immune system and local host cell response could be appropriate biomarkers, but their significance is currently unknown.

**Conclusions:** Conventional biomarkers for UTIs have low specificity. The use of urinary NGAL and interleukins could improve the sensitivity and specificity of laboratory diagnosis of UTIs.

## Summary of findings

1. Conventional biomarkers (pyuria, nitrite, proteinuria, CRP, PCT) have weak accuracy in general for diagnosis and differential diagnosis for UTIs.
2. The urothelium is an interactive immunological structure that produces an enormous quantity of molecules during the host-pathogene interaction. All of these molecules can form a subject for further research as a potential biomarker for UTIs.
3. Most data is collected on NGAL and interleukins, but their role is not exclusive in diagnostics considering the limiting factors and the lack of well-designed, confirming studies.
4. There are studies with at least 50 or more other biomarkers with either promising or controversial outcomes on their clinical usefulness, which we present briefly.
5. Not only for diagnosis but also for etiologic diagnosis there is a novel technology (NMR-spectrometry) with convincing, but few studies.
6. None of the presented biomarkers can render an exceptional or exclusive role in the field of diagnostics, thus further research is needed to identify suitable, reliable and easily measurable biomarkers.

## 1 Introduction

Urinary tract infections (UTIs) are amongst the most common infections worldwide. The diagnosis is based on symptoms, laboratory and imaging findings depending on the clinical manifestation. In this article, we will use the terminology as defined by the European Urological Association (EAU) in their guideline. For details, see Table 1 (Tab. 1).

Non-febrile, symptomatic lower UTIs react with local mucosal responses; febrile UTIs (f-UTIs)/acute pyelonephritis (APN) have an added significance to systemic host response; patients with asymptomatic bacteriuria (ABU) normally have no or only a discrete host response. Although these approaches provide an appropriate diagnosis in the majority of cases, their diagnostic accuracy or clinical usability can be limited in some situations, including UTIs in infants and in patients with neurogenic conditions or continuous catheterization.

Unfortunately, the widely used conventional biomarkers, including C-reactive protein (CRP), urine nitrite, leukocyte esterase, pyuria, and proteinuria, have a low sensitivity and specificity for predicting or differentiating UTIs, leading to over- or undertreatment. According to a retrospective pediatric study – analyzing 1,186 subjects, using these conventional diagnostic tools, 43% of the patients are overtreated, and 13% are undertreated, leading to recurrent infections, antibiotic resistance or kidney damage [1]. Thus, the need for a suitable, specific, easily measurable, widely available and quick biomarker for UTIs is in the focus of interest.

The search for the potential markers exploded when specific functions of the urothelium were described. The epithelial cells in bladder mucosa are not only a barrier, but also an active, responsive structure. During inflammatory setting, it produces cytokines in order to eliminate the microbial threat [2]. In this process, every produced molecule has its own role, forming a subject for identifying a suitable biomarker.

## 2 Methods

A literature search for biomarkers used in UTIs from January 1999 until May 2020 was performed. We used “urinary tract infections” and “biomarker” as the main key words in the PubMed, Medline and Cochrane databases. After reviewing, a total of 226 publications were identified, which were screened by title and abstract. Excluding the duplicates, we included 168 articles for full paper selection. Due to lacking data or weak study design, we excluded another 37, thus in the end, a total of 131 articles were included into this paper. Based on the results, we divided the available biomarkers into 5 groups:

1. conventional markers;
2. promising, thoroughly studied biomarkers;
3. promising biomarkers that need further studies;
4. biomarkers of unknown significance;
5. controversial, not useful markers.

Under the term “promising biomarkers”, we understand those markers that performed remarkable results on a scientific or on a statistical basis. For detailed information, see Table 2 (Tab. 2).

## 3 Results

Out of the 131 articles, 70 articles were on children, 49 on adults. The remaining 12 articles were either basic research or reviews. 55 studies operated with patient numbers over 50. In 30 articles, we found data on conventional biomarkers (CRP, procalcitonin (PCT), pyuria, nitrite etc.). These studies were either comparative with novel biomarkers or comparative with other conventional biomarkers. We did not deal with the latter ones, because our primary target was to identify and analyze novel biomarkers.

### 3.1 Conventional markers

In this category, we selected those markers that have been in clinical use from the beginning of laboratory diagnostics of UTIs.

Based on a retrospective study analyzing 1,223 cases, pyuria’s negative predictive value (NPV) is 75%, and the positive predictive value (PPV) is 40% in UTIs. Neither centrifugation nor staining the samples add any diagnostic advantage [3]. According to a review by Averbeck et al., a nitrite test is also a surrogate marker. However, its sensitivity is only 35–57%, but PPV is high with 96%. Combining pyuria with nitrite indicates 67% of the culture-positive cases [4]. Considering that proteinuria can occur in a series of other disease with a varying ratio of the excreted proteins, it is a non-specific marker of UTIs [5]. Lower UTIs induce the local immune system. It is obvious that acute phase proteins like CRP or PCT do not markedly elevate in serum. Based on a Cochrane meta-analysis, CRP has a sensitivity of 94%, and a specificity of 39%, indicating APN with the cut-off value of 2 mg/dl [6]. In a large, prospective study among children, PCT had 86% sensitivity and 89% specificity in detecting APN using 1.3 ng/ml as cut-off value [7].

### 3.2 Promising, thoroughly studied biomarkers

#### 3.2.1 Neutrophil gelatinase-associated lipocalin (NGAL)

We identified 49 articles on the following biomarkers, where studies with larger patient numbers (above 50 subjects) and/or with specific, well-defined, statistically proven outcomes were available. In specific patient groups, more difficult to study, as neurogenic bladder disorder and elderly patients with ABU, studies with smaller patient groups were also considered.

NGAL is an acute-phase protein [8], [9]. It was first observed in acute kidney injury affecting the proximal tubuli [10], [11]. It has a far-reaching effect on immune processes and has a direct bacteriostatic effect by blocking the siderophores on the Gram-negative bacterial wall [12]. NGAL levels elevate after 12 hours of the infection and reach their peak within 3 days [13]. Its secretion shows a variation by age and gender, but differences are small, thus there is no need for standardization [14]. NGAL excretion is independent from glomerular filtration, thus standardization for creatinine is not necessary [15]. Table 3 (Tab. 3) summarizes the results of studies on NGAL.

Based on the results, urinary NGAL can be a suitable marker for diagnosing UTIs in children and in adults. Although serum NGAL elevates during UTIs as well, referring to the listed data, urinary NGAL seems to be a more sensitive marker for predicting UTIs. For differential diagnosis of upper from lower UTIs, NGAL also could be a useful marker. Although the difference between the two groups was not significant in every study, a remarkable disparity could be observed between these groups [16]. Urine NGAL (U-NGAL) could be used for monitoring as well, because its level correlates with the duration of infection [17].

#### 3.2.2 Cytokines

During the host cell-pathogen interaction, a cytokine burst occurs in order to recruit the cells of the innate immune system and enhance the defense against pathogens. In UTIs, cytokines are mainly produced locally in the uroepithelial cell lining of the bladder and secreted into the urine. Generally, creatinine correlation of the cytokine content to compensate for different dilutions of the urine is not considered necessary [18]. Mostly, interleukin 1-beta (IL-1B), interIeukin-6 (IL-6) and interleukin-8 (IL-8) are studied. IL-1B could be a promising marker for differentiation between upper and lower UTIs [19]. Studies performed on different cytokines are shown in Table 4 (Tab. 4).

IL-6 and IL-8 are expressed rapidly after getting into contact with pathogens. IL-6 not only recruits immune cells, but also initiates gene cascades in order to produce antimicrobial peptides [20]. Due to its key role, it can be a predictor for UTIs and a marker of differentiation.

IL-8 has a central role in all inflammatory processes. Although its elevated concentration is observed in UTIs and can be a predictor of acute pyelonephritis, its specificity is low. It raises in every kind of congenital urinary anomaly, except antenatal renal pelvic dilatation. Thus, IL-8 is not suitable for diagnosing UTIs when an anatomical disorder is present [21].

In a recent observational study, 466 patients were enrolled. It turned out that measuring IL-8 in urine along with serum CRP and neutrophil-lymphocyte ratio (NLR) could indicate the pathogen among adults with type 2 diabetes mellitus. According to the authors, lower levels of CRP (median value: 33 mg/dl), higher levels of NLR (over 3.5) and higher levels of urine IL-8 (uIL-8) (median: 2,120 pg/ml) refer to extended spectrum beta-lactamase (ESBL) producing *Escherichia coli* infection. The opposite findings (median CRP under 39.8 mg/dl, NLR under 3.5, uIL-8 median 668 pg/ml) could indicate the presence of UTIs caused by ESBL producing *Klebsiella pneumoniae*. Increased NLR and IL-8 not only refers to the etiology, but also indicates the occurrence of renal damage. Among the aforementioned markers, NLR had the strongest predictive value for diagnosing UTIs caused by ESBL producing *E. coli* with 88% sensitivity, 73% specificity, 93% PPV and 24% NPV [22].

Using cytokines for diagnosing UTIs is limited by their low specificity. Elevated levels can be seen in a variety of other diseases, where the immune system is activated for a shorter or longer period [23]. A promising solution for this is to find cytokine combinations for a specific disease and develop cytokine pattern studies [24].

### 3.3 Promising biomarkers that need further studies

In this category, we listed biomarkers that have promising results, but due to small numbers of studies, data is lacking about their actual usefulness.

#### 3.3.1 Heparin binding protein (HBP)

HBP is released from activated neutrophils. HBP has a multiple role in inflammation [25]. On the one hand, it induces macromolecular efflux in endothels, thus responsible for swelling and edema forming. On the other hand, it is chemo-attractant and activates monocytes and macrophages [26], [27]. In UTIs, it has an excellent value not only in predicting, but also in differentiating upper from lower UTIs. For studies on HBP, see Table 5 (Tab. 5).

#### 3.3.2 Matrix metalloprotease-9 (MMP-9)

MMP-9 is a metalloprotease enzyme with a specific role in degradation of extracellular matrix components. During UTIs, MMP-9 often occurs in complex with NGAL [28]. Hatipoglu et al. measured the MMP-9/NGAL complex level in 145 childrens’ urine [29]. They found 98% sensitivity and 97% specificity with the cut-off value of 0.08 ng/mg for predicting the diagnosis of UTIs. It could be a better indicator than NGAL alone. Due to the high sensitivity and specificity, it could be used for distinguishing ABU from UTIs. Thanks to its kinetics, it is suitable for monitoring as well [29].

#### 3.3.3 Human neutrophil peptides-1,-3 (HNP-1,-3), human defensin-5 (HD-5)

Alpha defensins originate from white blood cells and epithelial cells. In a recent, observational, non-comparative study, using the cut-off values of 174 pg/mg for HD-5 and 383 μg/mg for HNP-1, the sensitivity was 86% and the specificity was 88% for predicting urine culture positivity among 199 children with UTIs [30]. There is a lack of further studies to determine the actual usefulness of these two markers.

#### 3.3.4 H1-nuclear magnetic resonance (H1-NMR) spectrometry

H_1_-NMR spectrometry is a novel laboratory method for detecting metabolites in various samples with limited accessibility. Most human diseases have characteristic modifications in the metabolite profile of fluids prior and during the development of clinical symptoms [31].

Searching a discriminative marker for bacterial UTIs, acetic acid occurred. Using 0.03 mmol/mmol as cut-off, it has 91% sensitivity and 95% specificity for predicting bacterial UTIs. 

Trymethylamine (TMA) is a natural metabolite of *E. coli* activity. With measuring the TMA/CRN ratio, the presence of an *E.coli* infection can be determined with 97% specificity and 66% sensitivity using the 0.0117 mmol/mmol value as cut-off. Results were based on analyzing 133 urine samples [31].

6-hydroxinicotinic acid (6-OHNA) is a metabolite of nicotinic acid, which is a metabolite of the *Pseudomonas aeruginosa* and *Klebsiella* species. With this method, etiological diagnosis could be made. Results were based on 30 samples [32].

#### 3.3.5 Lactoferrin (LF)

Lactoferrin is a stable iron-binding protein that circulates in serum. Using the 200 ng/ml as a cut-off, its specificity is 89% and its sensitivity 93% for distinguishing between ABU and UTIs [33]. These results originate from a comparative, observational study among adults. Although the study population was over 100 people, no one has confirmed the results ever since.

#### 3.3.6 Heat shock protein-70 (HSP-70)

HSPs upregulate during infection. For predicting UTIs, its sensitivity and specificity was 100% using the 158 µg/ml as cut-off in a prospective, comparative, observational study among 40 children with febrile UTIs [34].

#### 3.3.7 Bone morphogenic protein-2 (BMP-2) and cystatin C (CysC)

BMP-2 has a role in organ regeneration. Its level significantly increases in urinary stone formation and in infections.

Cystatin C is an endogenous biomarker for renal damage. It has a predictive value in diagnosing APN in children (cut-off: 1.08 mg/dl, sensitivity 58%, specificity 87%). According to a single, small observational study (45 patients), both markers have high values in diagnosing UTIs and predicting stone formation. Using 44 pg/ml as cut-off value for urinary BMP-2 for predicting UTIs has 92% sensitivity, 80% specificity and 86% accuracy. For urine CysC, the sensitivity was 85%, specificity 91% and accuracy 88%, using 525 ng/ml as cut-off [35].

#### 3.3.8 Lipopolysacharide-binding protein (LBP)

LBP is an acute phase protein. In a single, observational study among children, LBP had a sensitivity of 96%, and a specificity of 100% with an undetermined cut-off value [36]. Although the study population was over 100 children, data is lacking about its exact usefulness.

### 3.4 Biomarkers of unknown significance

In this category, we listed biomarkers that are elevated during UTIs, but there is insufficient information about their clinical usefulness (Table 6 (Tab. 6)).

### 3.5 Controversial, not useful markers

Under controversial biomarkers, we listed molecules where studies reported contradictory results regarding their usefulness for diagnosis or differential diagnosis of UTIs. The common feature in these studies were the small populations included.

Useless markers are not suitable for diagnosis or differential diagnosis in UTIs. Mostly, these biomarkers do not correlate with the course of the disease, or the measurement is technically too complicated for daily application.

The following markers were either not useful for predicting UTIs or not practical: copeptin, KIM-1, ATP, TREM-1, apolipoprotein D, alpha amylase 2B, non-secretory ribonuclease, fibronectin, elastase, etc. [17], [37], [38], [39], [40].

Controversial data on their usefulness can be found for the following biomarkers: Beta-2-microglobulin, pentraxin-3, prosaposin, Tamm-Horsfall Protein, Arginin-Vasopressin [37], [41], [42].

## 4 Discussion

UTIs are common infections in every age group. Under special circumstances, timely diagnosis might be challenging. In these situations, clinicians must decide on laboratory findings, which have a weak accuracy in general. As seen in the details, none of the aforementioned biomarkers can predict the presence of a UTI exclusively. Thus, the interest turns to novel markers, specifically in special patients populations, for example children, who cannot describe their symptoms reliably, or among patients with constant catheterization, who have asymptomatic bacteriuria with or without specific symptoms referring to infection. In these cases, measuring the severity of the immune response could be a suitable alternative to decide about the need of antibiotic treatment. In addition, the diagnostic accuracy of biomarkers can probably only be determined when combined with a detailed and standardized scoring of subjective symptoms along severity indexing and individual risk ratification. For this reason, using validated questionnaires, for example the Acute Cystitis Symptom Score questionnaire [43], [44], could allow stratification of different clinical scenarios in combination with novel biomarkers in the future.

There are specific difficulties in each patient population in which UTI biomarkers are needed, but unfortunately, the patient groups with the highest need for a biomarker are also the ones that are most difficult to study. Not only symptom scoring is difficult; when ABU is frequent, the host response differentiation is obstructed. Furthermore, the comparator, ideally intraindividual comparison before and after a UTI episode, is commonly replaced by matched controls. In our literature search, we found that approximately one third of the articles were on NGAL or on cytokines. Considering that cytokines are key components of the local host-cell response, these are promising biomarkers for differentiating upper from lower UTIs – especially IL-6 and IL-8 [18], [21]. Furthermore, they might be useful in measuring the level of local immune response during uncomplicated lower UTIs, which can help the decision about non-antibiotic treatment [45].

NGAL also has a potential in the diagnosis of UTIs with high sensitivity and specificity. However, an important limitation is that it is a common marker of kidney injury, thus acute or chronic disease and other potential conditions that are yet unknown can elevate its level along with an infectious disease. In this field, further observations are needed [16].

Most of the published data on biomarkers focus on children. There were examples when the context were proven for adults as well, thus we believe that further findings should be valid for adults too. However, due to a lack of studies, the probative value is still missing [16]. Another weakness of the literature search is the lack of data on men, as well as on other forms of UTIs.

Finding suitable biomarkers for UTIs has limitations: The immune system is constantly influenced by varying conditions (chronic, metabolic or malignant disease, intercurrent infections etc.) which may influence the expression of biomarkers. Further research could include a search for bacterial metabolites or volatile organic compounds in urine [31], [44]. The amount of the novel biomarkers is enormous. Therefore, we here focused on those that have promising results or were studied in larger quantities. The cut-off values varied in a wide range in the different studies. The production of these molecules may vary by the severity of the symptoms, the activation of the immune system, anatomic anomalies etc. In order to determine precise cut-off values, a meta-analysis should be performed. However, due to the large number of variables involved, it is uncertain whether such an analysis could be conducted successfully.

## 5 Further research

Each of the biomarkers discussed above needs to be investigated and validated further in well designed clinical studies to determine their clinical usefulness for diagnosis and treatment outcome in patients with different categories of UTI, including uncomplicated and complicated UTI and urosepsis.

## 6 Conclusion

Since the diagnostic accuracy and clinical usability of traditional biomarkers for UTIs in certain clinical settings are limited, there is a need for novel, accurate and widely available biomarkers. Urinary NGAL and interleukins are the most suitable biomarkers that are currently available for clinicians with a potential to improve the sensitivity and specificity of laboratory diagnosis of UTIs.

## Abbreviations

- **6-OHNA:** 6’-hydroxy nicotinic acid
- **8-oxodG:** 8’-hydroxy-2’-deoxyguanine
- **ABU:** Asymptomatic bacteriuria
- **APN:** Acute pyelonephritis
- **ATP:** Adenosine triphosphate
- **BMP-2:** Bone morphogenic protein-2
- **CA19-9:** Carboanhydrate antigene 19-9
- **CAP37:** Cationic antimicrobial protein of 37 kd (synonyme for HPB)
- **CD14:** Cluster of differentiation 14
- **CD44:** Cluster of differentiation 44
- **CIC:** Clean intermittent catheterisation
- **CRN:** Creatinine
- **CRP:** C-reactive protein
- **CysC:** Cystatin C
- **ESBL:** Extended spectrum beta lactamase
- **H1-NMR:** H_1_-nuclear magnetic resonance
- **HBP:** Heparin binding protein
- **HD-5:** Human defensin-5
- **HNP-1:** Human neutrophil peptide-1
- **HNP-3:** Human neutrophil peptide-3
- **HSP-70:** Heat shock protein-70
- **IL:** Interleukin
- **IL-1B:** Interleukin-1 beta
- **IL-6:** Interleukin-6
- **IL-8:** Interleukin-8
- **LBP:** Lipopolisaccharide binding protein
- **LF:** Lactoferrin
- **l-UTI:** Lower urinary tract infection
- **NGAL:** Neutrophil gelatinase-associated lipocalin
- **NLR:** Neutrophil-lymphocyte Ratio
- **NPV:** Negative predictive value
- **MCP-1:** Monocyte chemotactic protein-1
- **MMP-9:** Matrix metalloprotease-9
- **PCT:** Procalcitonin
- **PGE2:** Prostaglandin-E2
- **PPV:** Positive predictive value
- **sIL-1B:** Serum interleukin 1 beta
- **sIL-6:** Serum interleukin-6
- **sIL-8:** Serum interleukin-8
- **s-NGAL:** Serum neutrophil gelatinase-associated lipocalin
- **s-UTI:** Symptomatic urinary tract infection
- **TAC:** Total antioxidant capacity
- **TMA:** Trimethylamine
- **TLR:** Toll-like receptor
- **TREM-1:** Triggering receptor expressed on myeloid cells-1
- **uIL-1B:** Urinary interleukin-1 beta
- **uIL-6:** Urinary interleukin-6
- **uIL-8:** Urinary IL-8
- **u-NGAL:** Urinary neutrophil gelatinase-associated lipocalin
- **UPJO:** Uretero-pelvic junction obstruction
- **UTI:** Urinary tract infection
- **u-UTI:** Upper urinary tract infection
- **YKL-40:** Tyrosine (Y) – lysine (K) – leucin (L) of 40 kDa
- **VUR:** Vesico-ureteral reflux

## Note

This article is also to be published as a chapter of the Living Handbook „Urogenital Infections and Inflammations“ [46].

## Competing interests

The authors declare that they have no competing interests.

## Figures and Tables

**Table 1:**
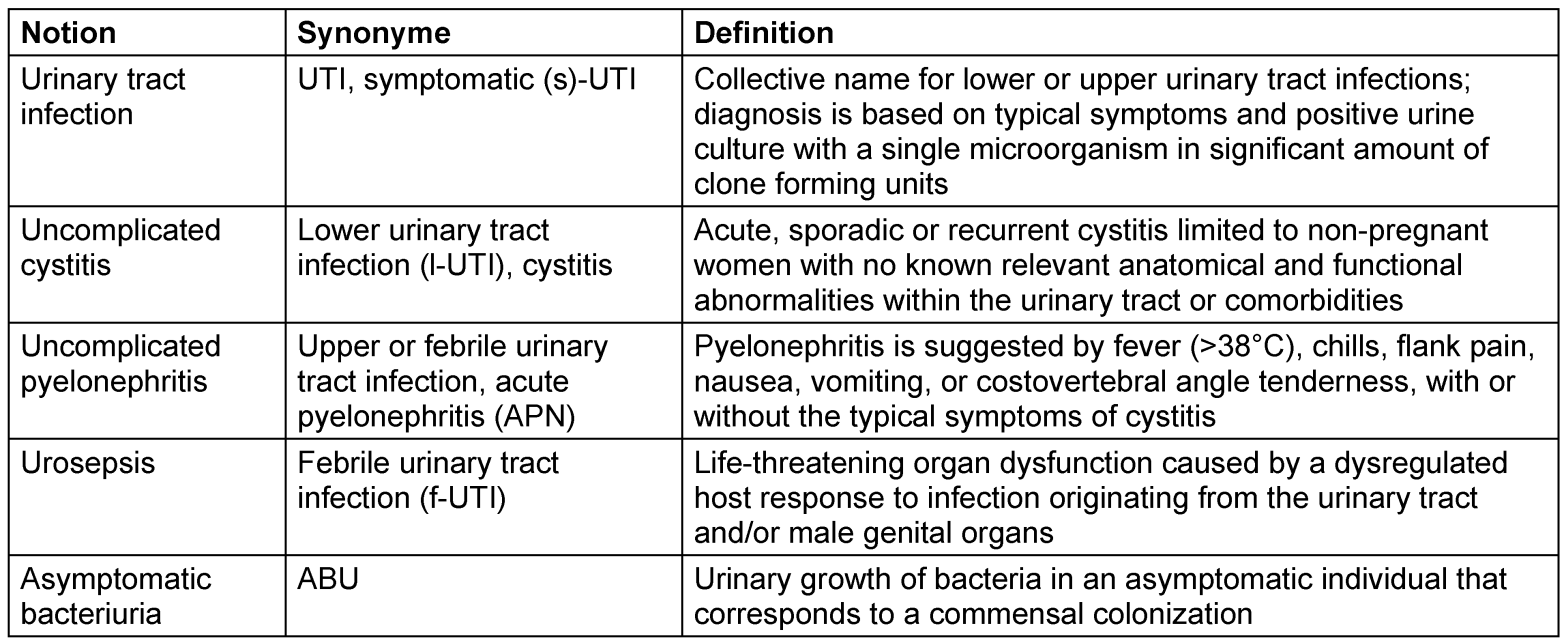
Notions and definitions used in the article

**Table 2:**
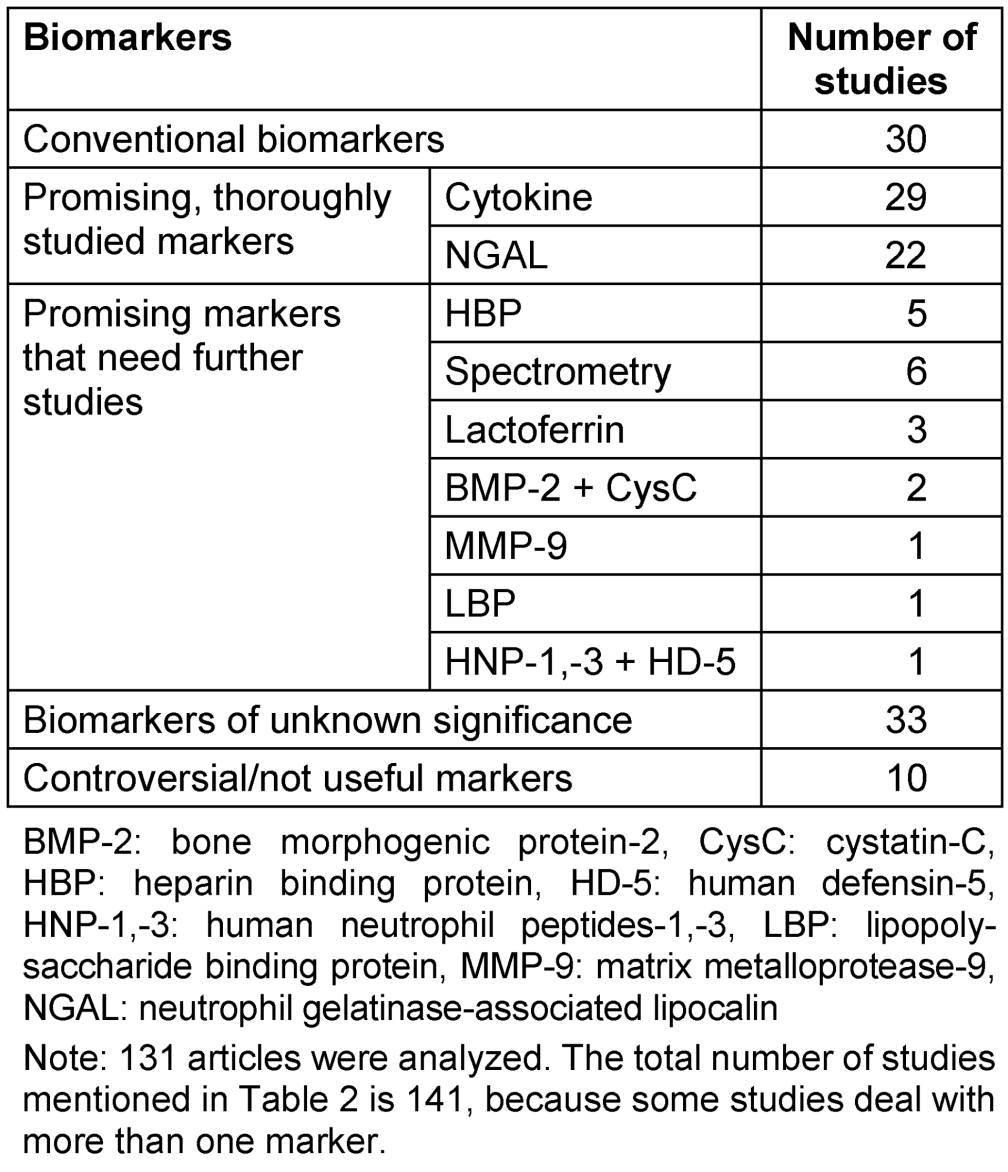
Identified studies on different biomarkers

**Table 3:**
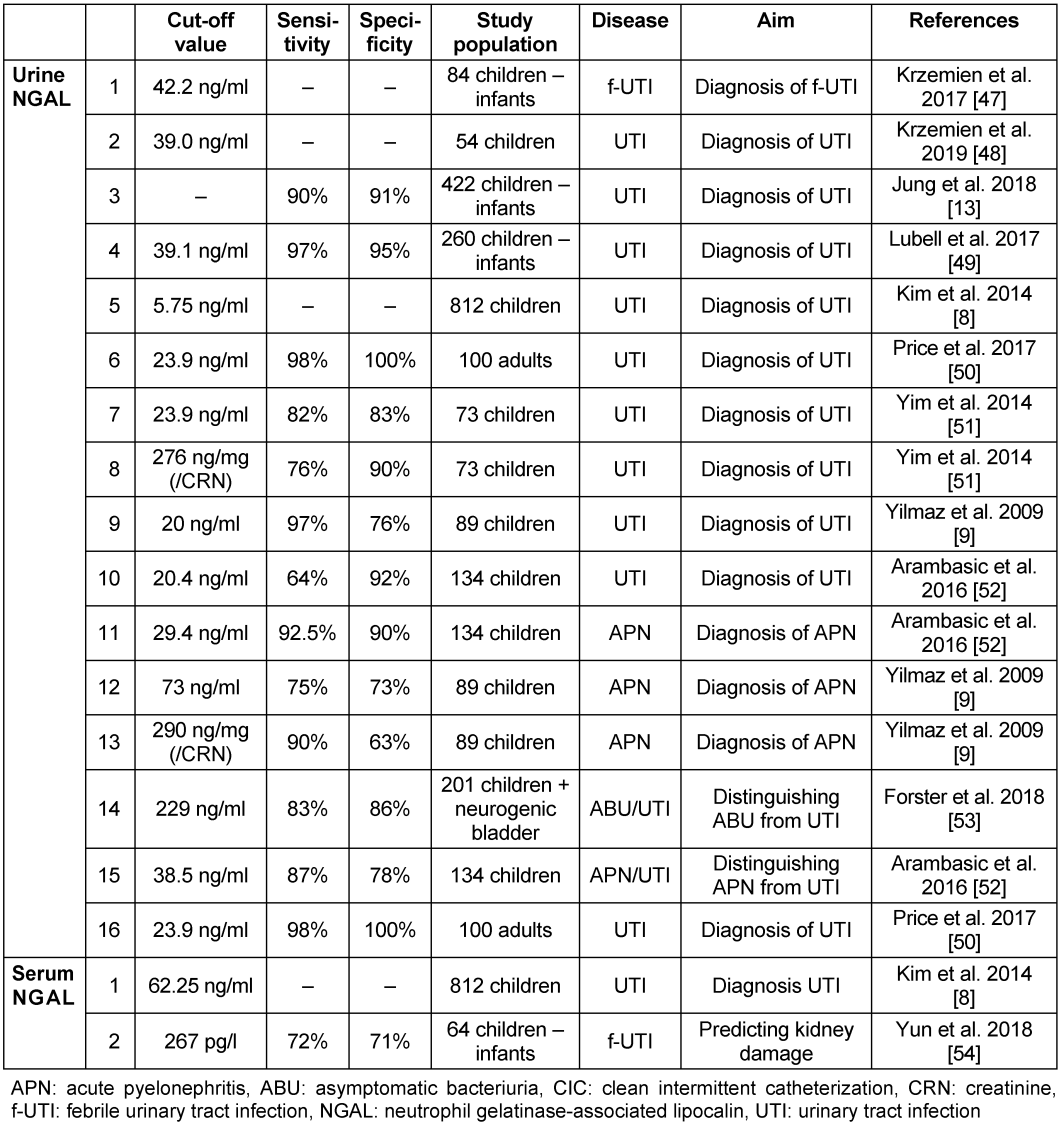
Urinary and serum NGAL for predicting UTI and differentiating infections

**Table 4:**
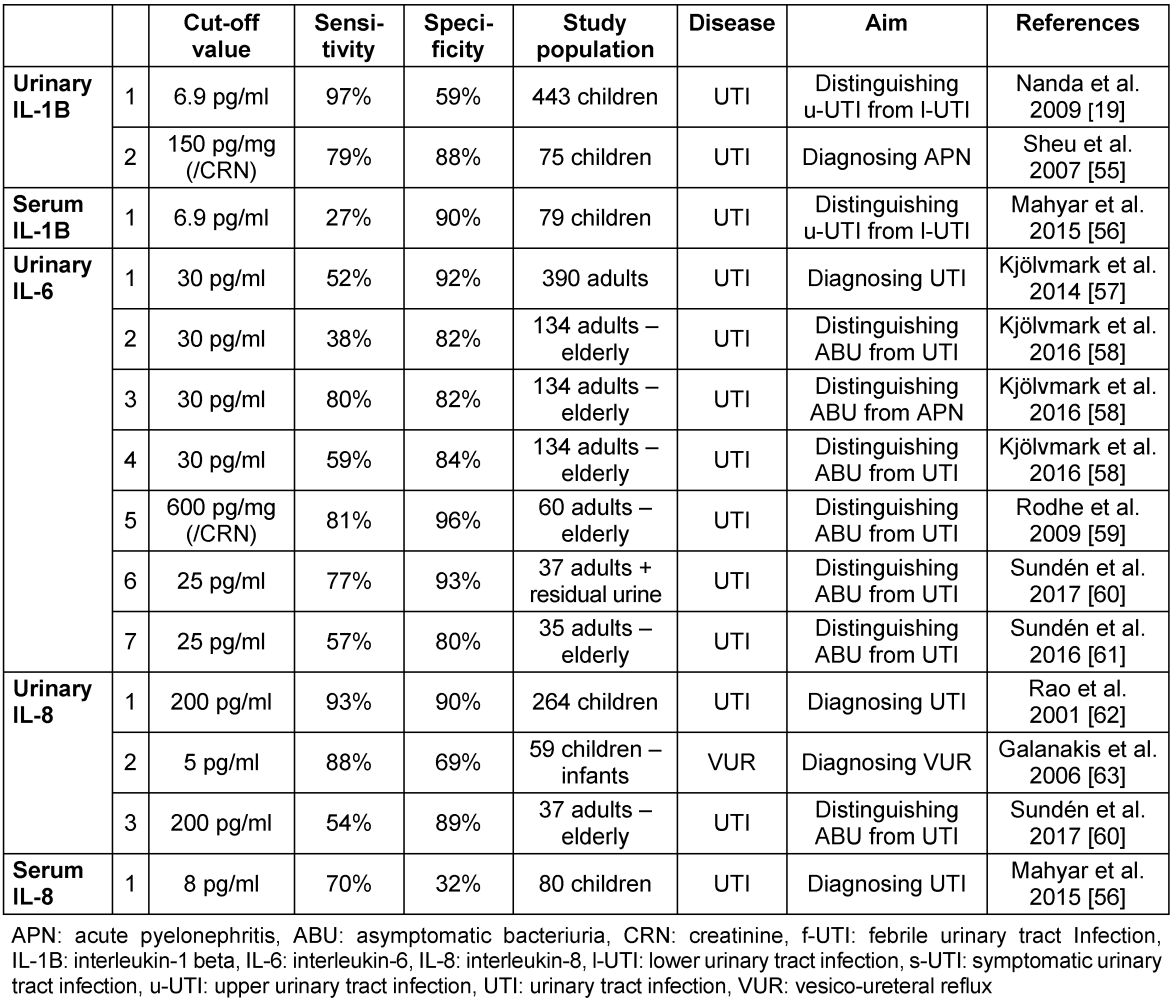
Diagnostic and differential diagnostic value of Il-1B, IL-6, IL-8

**Table 5:**
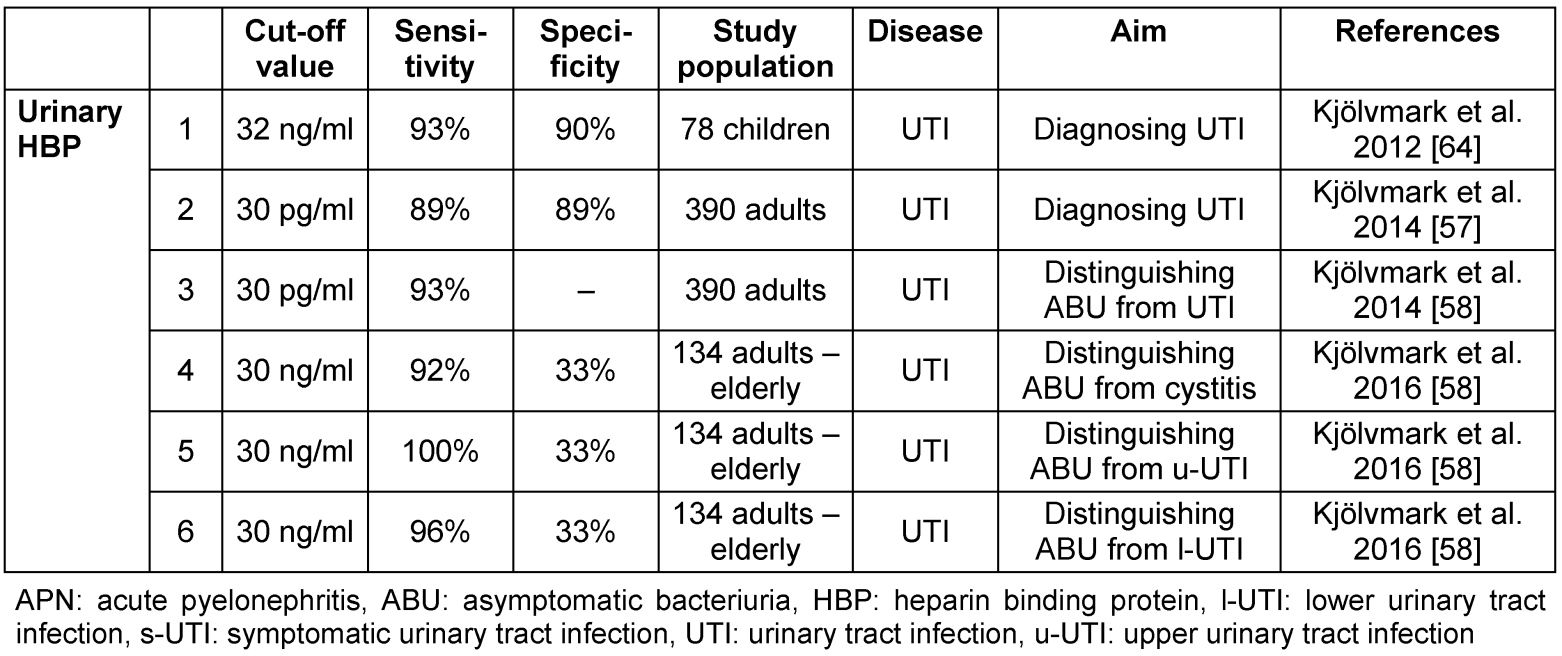
Studies on HBP

**Table 6:**
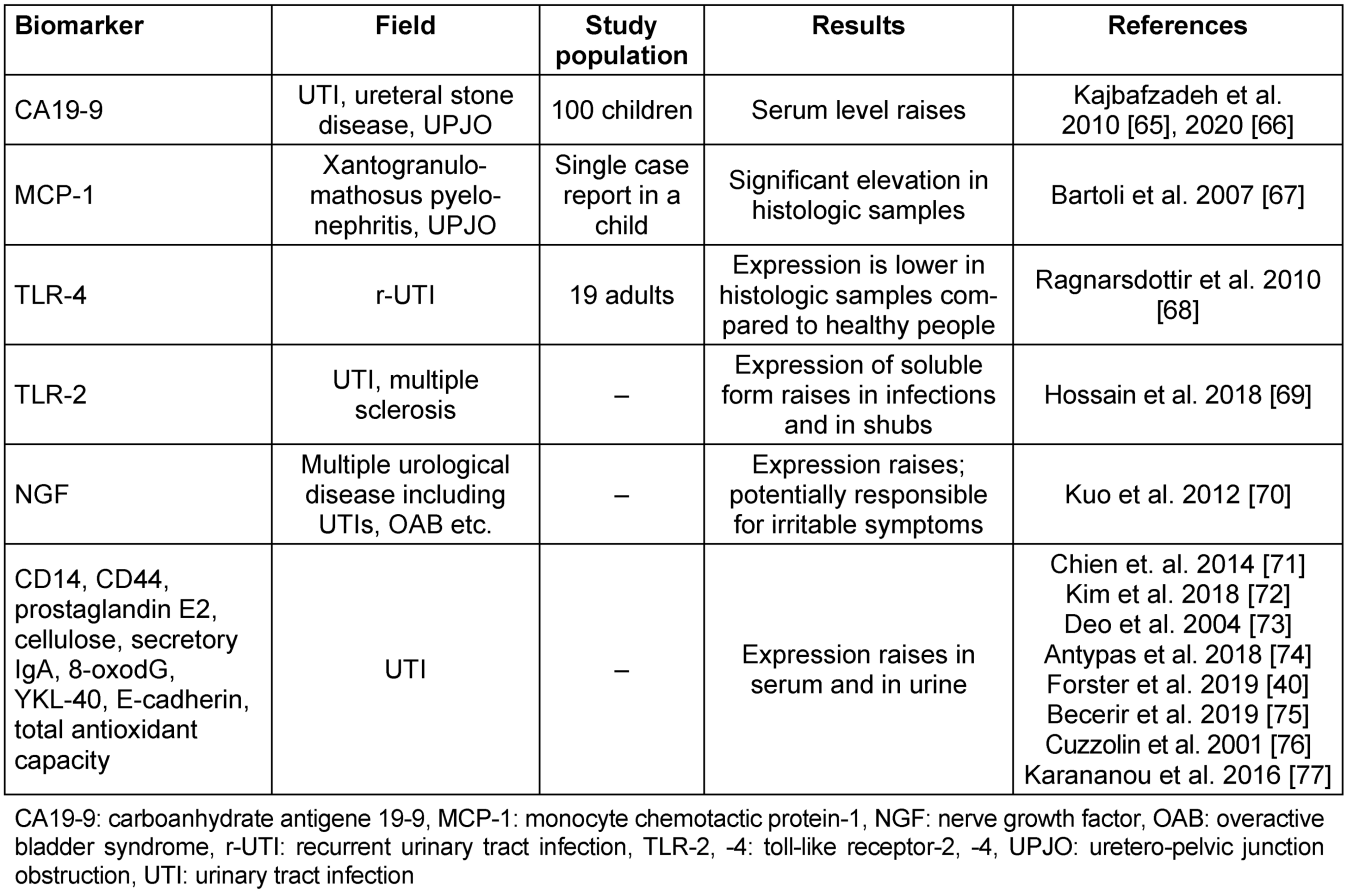
Summary of biomarkers of unknown significance

## References

[1] Hannula A, Perhomaa M, Venhola M, Pokka T, Renko M, Uhari M (2012). Long-term follow-up of patients after childhood urinary tract infection. Arch Pediatr Adolesc Med.

[2] Yeh J, Lu M, Alvarez-Lugo L, Chai TC (2019). Bladder urothelial BK channel activity is a critical mediator for innate immune response in urinary tract infection pathogenesis. Am J Physiol Renal Physiol.

[3] Kupelian AS, Horsley H, Khasriya R, Amussah RT, Badiani R, Courtney AM, Chandhyoke NS, Riaz U, Savlani K, Moledina M, Montes S, O'Connor D, Visavadia R, Kelsey M, Rohn JL, Malone-Lee J (2013). Discrediting microscopic pyuria and leucocyte esterase as diagnostic surrogates for infection in patients with lower urinary tract symptoms: results from a clinical and laboratory evaluation. BJU Int.

[4] Averbeck MA, Rantell A, Ford A, Kirschner-Hermanns R, Khullar V, Wagg A, Cardozo L (2018). Current controversies in urinary tract infections: ICI-RS 2017. Neurourol Urodyn.

[5] Andersson L, Preda I, Hahn-Zoric M, Hanson LA, Jodal U, Sixt R, Barregard L, Hansson S (2009). Urinary proteins in children with urinary tract infection. Pediatr Nephrol.

[6] Shaikh N, Borrell JL, Evron J, Leeflang MM (2015). Procalcitonin, C-reactive protein, and erythrocyte sedimentation rate for the diagnosis of acute pyelonephritis in children. Cochrane Database Syst Rev.

[7] Julián-Jiménez A, Gutiérrez-Martín P, Lizcano-Lizcano A, López-Guerrero MA, Barroso-Manso Á, Heredero-Gálvez E (2015). Usefulness of procalcitonin and C-reactive protein for predicting bacteremia in urinary tract infections in the emergency department. Actas Urol Esp.

[8] Kim BH, Yu N, Kim HR, Yun KW, Lim IS, Kim TH, Lee MK (2014). Evaluation of the optimal neutrophil gelatinase-associated lipocalin value as a screening biomarker for urinary tract infections in children. Ann Lab Med.

[9] Yilmaz A, Sevketoglu E, Gedikbasi A, Karyagar S, Kiyak A, Mulazimoglu M, Aydogan G, Ozpacaci T, Hatipoglu S (2009). Early prediction of urinary tract infection with urinary neutrophil gelatinase associated lipocalin. Pediatr Nephrol.

[10] Singer E, Markó L, Paragas N, Barasch J, Dragun D, Müller DN, Budde K, Schmidt-Ott KM (2013). Neutrophil gelatinase-associated lipocalin: pathophysiology and clinical applications. Acta Physiol (Oxf).

[11] Hirsch R, Dent C, Pfriem H, Allen J, Beekman RH, Ma Q, Dastrala S, Bennett M, Mitsnefes M, Devarajan P (2007). NGAL is an early predictive biomarker of contrast-induced nephropathy in children. Pediatr Nephrol.

[12] Nasioudis D, Witkin SS (2015). Neutrophil gelatinase-associated lipocalin and innate immune responses to bacterial infections. Med Microbiol Immunol.

[13] Jung N, Byun HJ, Park JH, Kim JS, Kim HW, Ha JY (2018). Diagnostic accuracy of urinary biomarkers in infants younger than 3 months with urinary tract infection. Korean J Pediatr.

[14] Forster CS, Johnson K, Patel V, Wax R, Rodig N, Barasch J, Bachur R, Lee RS (2017). Urinary NGAL deficiency in recurrent urinary tract infections. Pediatr Nephrol.

[15] Mori K, Lee HT, Rapoport D, Drexler IR, Foster K, Yang J, Schmidt-Ott KM, Chen X, Li JY, Weiss S, Mishra J, Cheema FH, Markowitz G, Suganami T, Sawai K, Mukoyama M, Kunis C, D'Agati V, Devarajan P, Barasch J (2005). Endocytic delivery of lipocalin-siderophore-iron complex rescues the kidney from ischemia-reperfusion injury. J Clin Invest.

[16] Urbschat A, Obermüller N, Paulus P, Reissig M, Hadji P, Hofmann R, Geiger H, Gauer S (2014). Upper and lower urinary tract infections can be detected early but not be discriminated by urinary NGAL in adults. Int Urol Nephrol.

[17] Petrovic S, Bogavac-Stanojevic N, Peco-Antic A, Ivanisevic I, Kotur-Stevuljevic J, Paripovic D, Sopic M, Jelic-Ivanovic Z (2013). Clinical application neutrophil gelatinase-associated lipocalin and kidney injury molecule-1 as indicators of inflammation persistence and acute kidney injury in children with urinary tract infection. Biomed Res Int.

[18] Sundvall PD, Elm M, Ulleryd P, Mölstad S, Rodhe N, Jonsson L, Andersson B, Hahn-Zoric M, Gunnarsson R (2014). Interleukin-6 concentrations in the urine and dipstick analyses were related to bacteriuria but not symptoms in the elderly: a cross sectional study of 421 nursing home residents. BMC Geriatr.

[19] Nanda N, Juthani-Mehta M (2009). Novel biomarkers for the diagnosis of urinary tract infection-a systematic review. Biomark Insights.

[20] Ching CB, Gupta S, Li B, Cortado H, Mayne N, Jackson AR, McHugh KM, Becknell B (2018). Interleukin-6/Stat3 signaling has an essential role in the host antimicrobial response to urinary tract infection. Kidney Int.

[21] Bitsori M, Karatzi M, Dimitriou H, Christakou E, Savvidou A, Galanakis E (2011). Urine IL-8 concentrations in infectious and non-infectious urinary tract conditions. Pediatr Nephrol.

[22] Saheb Sharif-Askari F, Saheb Sharif-Askari N, Guella A, Alabdullah A, Bashar Al Sheleh H, Maher Hoory AlRawi A, Sami Haddad E, Hamid Q, Halwani R, Hamoudi R (2020). Blood Neutrophil-to-Lymphocyte Ratio and Urine IL-8 Levels Predict the Type of Bacterial Urinary Tract Infection in Type 2 Diabetes Mellitus Patients. Infect Drug Resist.

[23] Ghoniem G, Faruqui N, Elmissiry M, Mahdy A, Abdelwahab H, Oommen M, Abdel-Mageed AB (2011). Differential profile analysis of urinary cytokines in patients with overactive bladder. Int Urogynecol J.

[24] Shaikh N, Martin JM, Hoberman A, Skae M, Milkovich L, McElheny C, Hickey RW, Gabriel LV, Kearney DH, Majd M, Shalaby-Rana E, Tseng G, Kolls J, Horne W, Huo Z, Shope TR (2020). Biomarkers that differentiate false positive urinalyses from true urinary tract infection. Pediatr Nephrol.

[25] Tapper H, Karlsson A, Mörgelin M, Flodgaard H, Herwald H (2002). Secretion of heparin-binding protein from human neutrophils is determined by its localization in azurophilic granules and secretory vesicles. Blood.

[26] Gautam N, Olofsson AM, Herwald H, Iversen LF, Lundgren-Akerlund E, Hedqvist P, Arfors KE, Flodgaard H, Lindbom L (2001). Heparin-binding protein (HBP/CAP37): a missing link in neutrophil-evoked alteration of vascular permeability. Nat Med.

[27] Soehnlein O, Lindbom L (2009). Neutrophil-derived azurocidin alarms the immune system. J Leukoc Biol.

[28] Parks WC, Wilson CL, López-Boado YS (2004). Matrix metalloproteinases as modulators of inflammation and innate immunity. Nat Rev Immunol.

[29] Hatipoglu S, Sevketoglu E, Gedikbasi A, Yilmaz A, Kiyak A, Mulazimoglu M, Aydogan G, Ozpacaci T (2011). Urinary MMP-9/NGAL complex in children with acute cystitis. Pediatr Nephrol.

[30] Watson JR, Hains DS, Cohen DM, Spencer JD, Kline JM, Yin H, Schwaderer AL (2016). Evaluation of novel urinary tract infection biomarkers in children. Pediatr Res.

[31] Lussu M, Camboni T, Piras C, Serra C, Del Carratore F, Griffin J, Atzori L, Manzin A (2017). H NMR spectroscopy-based metabolomics analysis for the diagnosis of symptomatic E. coli-associated urinary tract infection (UTI). BMC Microbiol.

[32] Gupta A, Dwivedi M, Nagana Gowda GA, Ayyagari A, Mahdi AA, Bhandari M, Khetrapal CL (2005). (1)H NMR spectroscopy in the diagnosis of Pseudomonas aeruginosa-induced urinary tract infection. NMR Biomed.

[33] Arao S, Matsuura S, Nonomura M, Miki K, Kabasawa K, Nakanishi H (1999). Measurement of urinary lactoferrin as a marker of urinary tract infection. J Clin Microbiol.

[34] Yilmaz A, Yildirim ZY, Emre S, Gedikbasi A, Yildirim T, Dirican A, Ucar EO (2016). Urine heat shock protein 70 levels as a marker of urinary tract infection in children. Pediatr Nephrol.

[35] Salama RH, Alghasham A, Mostafa MS, El-Moniem AE (2012). Bone morphogenetic protein-2 will be a novel biochemical marker in urinary tract infections and stone formation. Clin Biochem.

[36] Tsalkidou EA, Roilides E, Gardikis S, Trypsianis G, Kortsaris A, Chatzimichael A, Tentes I (2013). Lipopolysaccharide-binding protein: a potential marker of febrile urinary tract infection in childhood. Pediatr Nephrol.

[37] Masajtis-Zagajewska A, Kurnatowska I, Wajdlich M, Nowicki M (2015). Utility of copeptin and standard inflammatory markers in the diagnostics of upper and lower urinary tract infections. BMC Urol.

[38] Gill K, Horsley H, Kupelian AS, Baio G, De Iorio M, Sathiananamoorthy S, Khasriya R, Rohn JL, Wildman SS, Malone-Lee J (2015). Urinary ATP as an indicator of infection and inflammation of the urinary tract in patients with lower urinary tract symptoms. BMC Urol.

[39] Sierra-Diaz E, Bravo Cuéllar A, Ortiz Lazareno PC, García Gutiérrez M, Georgina HF, Anaya Prado R (2017). Urine TREM-1 as a marker of urinary tract infection in children. J Int Med Res.

[40] Forster CS, Haffey WD, Bennett M, Greis KD, Devarajan P (2019). Identification of Urinary CD44 and Prosaposin as Specific Biomarkers of Urinary Tract Infections in Children With Neurogenic Bladders. Biomark Insights.

[41] Sandberg T, Bergmark J, Hultberg B, Jagenburg R, Trollfors B (1986). Diagnostic potential of urinary enzymes and beta 2-microglobulin in acute urinary tract infection. Acta Med Scand.

[42] Itoh Y (2002). [Current topics on urinary proteins: human albumin, protein 1, beta 2-microglobulin, and type IV collagen]. Rinsho Byori.

[43] Alidjanov JF, Naber KG, Pilatz A, Radzhabov A, Zamuddinov M, Magyar A, Tenke P, Wagenlehner FM (2020). Evaluation of the draft guidelines proposed by EMA and FDA for the clinical diagnosis of acute uncomplicated cystitis in women. World J Urol.

[44] Alidjanov JF, Naber KG, Pilatz A, Radzhabov A, Zamuddinov M, Magyar A, Tenke P, Wagenlehner FM (2020). Additional assessment of Acute Cystitis Symptom Score questionnaire for patient-reported outcome measure in female patients with acute uncomplicated cystitis: part II. World J Urol.

[45] Wawrysiuk S, Naber K, Rechberger T, Miotla P (2019). Prevention and treatment of uncomplicated lower urinary tract infections in the era of increasing antimicrobial resistance-non-antibiotic approaches: a systemic review. Arch Gynecol Obstet.

[46] Horváth J, Wullt B, Naber KG, Köves B, Bjerklund Johansen TE, Wagenlehner FME, Cho YH, Matsumoto T, Krieger JN, Shoskes D, Naber KG (2017-). Biomarkers in urinary tract infections – which ones are suitable for diagnostics and follow-up?. Urogenital Infections and Inflammations.

[47] Krzemień G, Pańczyk-Tomaszewska M, Adamczuk D, Kotuła I, Demkow U, Szmigielska A (2018). Neutrophil Gelatinase-Associated Lipocalin: A Biomarker for Early Diagnosis of Urinary Tract Infections in Infants. Adv Exp Med Biol.

[48] Krzemień G, Pańczyk-Tomaszewska M, Kotuła I, Demkow U, Szmigielska A (2019). Diagnostic accuracy of urine neutrophil gelatinase-associated lipocalin and urine kidney injury molecule-1 as predictors of acute pyelonephritis in young children with febrile urinary tract infection. Cent Eur J Immunol.

[49] Lubell TR, Barasch JM, Xu K, Ieni M, Cabrera KI, Dayan PS (2017). Urinary Neutrophil Gelatinase-Associated Lipocalin for the Diagnosis of Urinary Tract Infections. Pediatrics.

[50] Price JR, Guran L, Lim JY, Megli CJ, Clark AL, Edwards SR, Denman MA, Gregory WT (2017). Neutrophil Gelatinase-Associated Lipocalin Biomarker and Urinary Tract Infections: A Diagnostic Case-Control Study (NUTI Study). Female Pelvic Med Reconstr Surg.

[51] Yim HE, Yim H, Bae ES, Woo SU, Yoo KH (2014). Predictive value of urinary and serum biomarkers in young children with febrile urinary tract infections. Pediatr Nephrol.

[52] Arambašić J, Mandić S, Debeljak Ž, Mandić D, Horvat V, Šerić V (2016). Differentiation of acute pyelonephritis from other febrile states in children using urinary neutrophil gelatinase-associated lipocalin (uNGAL). Clin Chem Lab Med.

[53] Forster CS, Jackson E, Ma Q, Bennett M, Shah SS, Goldstein SL (2018). Predictive ability of NGAL in identifying urinary tract infection in children with neurogenic bladders. Pediatr Nephrol.

[54] Yun BA, Yang EM, Kim CJ (2018). Plasma Neutrophil Gelatinase-Associated Lipocalin as a Predictor of Renal Parenchymal Involvement in Infants With Febrile Urinary Tract Infection: A Preliminary Study. Ann Lab Med.

[55] Sheu JN, Chen MC, Cheng SL, Lee IC, Chen SM, Tsay GJ (2007). Urine interleukin-1beta in children with acute pyelonephritis and renal scarring. Nephrology (Carlton).

[56] Mahyar A, Ayazi P, Yarigarravesh MH, Khoeiniha MH, Oveisi S, Sahmani AA, Esmaeily S (2015). Serum interleukin -8 is not a reliable marker for prediction of vesicoureteral reflux in children with febrile urinary tract infection. Int Braz J Urol.

[57] Kjölvmark C, Påhlman LI, Åkesson P, Linder A (2014). Heparin-binding protein: a diagnostic biomarker of urinary tract infection in adults. Open Forum Infect Dis.

[58] Kjölvmark C, Tschernij E, Öberg J, Påhlman LI, Linder A, Åkesson P (2016). Distinguishing asymptomatic bacteriuria from urinary tract infection in the elderly - the use of urine levels of heparin-binding protein and interleukin-6. Diagn Microbiol Infect Dis.

[59] Rodhe N, Löfgren S, Strindhall J, Matussek A, Mölstad S (2009). Cytokines in urine in elderly subjects with acute cystitis and asymptomatic bacteriuria. Scand J Prim Health Care.

[60] Sundén F, Butler D, Wullt B (2017). Triggered Urine Interleukin-6 Correlates to Severity of Symptoms in Nonfebrile Lower Urinary Tract Infections. J Urol.

[61] Sundén F, Wullt B (2016). Predictive value of urinary interleukin-6 for symptomatic urinary tract infections in a nursing home population. Int J Urol.

[62] Rao WH, Evans GS, Finn A (2001). The significance of interleukin 8 in urine. Arch Dis Child.

[63] Galanakis E, Bitsori M, Dimitriou H, Giannakopoulou C, Karkavitsas NS, Kalmanti M (2006). Urine interleukin-8 as a marker of vesicoureteral reflux in infants. Pediatrics.

[64] Kjölvmark C, Akesson P, Linder A (2012). Elevated urine levels of heparin-binding protein in children with urinary tract infection. Pediatr Nephrol.

[65] Kajbafzadeh AM, Elmi A, Talab SS, Emami H, Esfahani SA, Saeedi P (2010). Urinary and serum carbohydrate antigen 19-9 as a biomarker in ureteropelvic junction obstruction in children. J Urol.

[66] Kajbafzadeh AM, Ladi Seyedian SS, Kameli SM, Nabavizadeh B, Boroomand M, Moghtaderi M (2020). Urinary carbohydrate antigen 19-9 level as a biomarker in children with acute pyelonephritis. Eur J Pediatr.

[67] Bartoli F, Gesualdo L, Niglio F, Gentile O, Penza R, Leggio S, Lasalandra C, Campanella V, Magaldi S (2007). Xanthogranulomatous pyelonephritis is associated with higher tissue expression of monocyte chemotactic protein-1. Eur J Pediatr Surg.

[68] Ragnarsdóttir B, Jönsson K, Urbano A, Grönberg-Hernandez J, Lutay N, Tammi M, Gustafsson M, Lundstedt AC, Leijonhufvud I, Karpman D, Wullt B, Truedsson L, Jodal U, Andersson B, Svanborg C (2010). Toll-like receptor 4 promoter polymorphisms: common TLR4 variants may protect against severe urinary tract infection. PLoS One.

[69] Hossain MJ, Morandi E, Tanasescu R, Frakich N, Caldano M, Onion D, Faraj TA, Erridge C, Gran B (2018). The Soluble Form of Toll-Like Receptor 2 Is Elevated in Serum of Multiple Sclerosis Patients: A Novel Potential Disease Biomarker. Front Immunol.

[70] Kuo HC (2012). Potential Biomarkers Utilized to Define and Manage Overactive Bladder Syndrome. Low Urin Tract Symptoms.

[71] Chien JW, Wang LY, Cheng YS, Tsai YG, Liu CS (2014). Urinary 8-hydroxy-2'-deoxyguanosine (8-oxodG) level can predict acute renal damage in young children with urinary tract infection. Biomarkers.

[72] Kim HH, Chung MH, Bin JH, Cho KS, Lee J, Suh JS (2018). Urinary YKL-40 as a Candidate Biomarker for Febrile Urinary Tract Infection in Young Children. Ann Lab Med.

[73] Deo SS, Vaidya AK (2004). Elevated levels of secretory immunoglobulin A (sIgA) in urinary tract infections. Indian J Pediatr.

[74] Antypas H, Choong FX, Libberton B, Brauner A, Richter-Dahlfors A (2018). Rapid diagnostic assay for detection of cellulose in urine as biomarker for biofilm-related urinary tract infections. NPJ Biofilms Microbiomes.

[75] Becerir T, Yüksel S, Evrengül H, Ergin A, Enli Y (2019). Urinary excretion of pentraxin-3 correlates with the presence of renal scar following acute pyelonephritis in children. Int Urol Nephrol.

[76] Cuzzolin L, Mangiarotti P, Fanos V (2001). Urinary PGE(2) concentrations measured by a new EIA method in infants with urinary tract infections or renal malformations. Prostaglandins Leukot Essent Fatty Acids.

[77] Karananou P, Fleva A, Tramma D, Alataki A, Pavlitou-Tsiontsi A, Emporiadou-Peticopoulou M, Papadopoulou-Alataki E (2016). Altered Expression of TLR2 and TLR4 on Peripheral CD14+ Blood Monocytes in Children with Urinary Tract Infection. Biomed Res Int.

